# Editorial: Formation of immunological niches in tumor microenvironments: mechanisms and therapeutic potential

**DOI:** 10.3389/fonc.2025.1675697

**Published:** 2025-09-09

**Authors:** Zilu Chen, Qingyu Luo, Weiling Li, Ying Luo, Xiaosheng Tan

**Affiliations:** ^1^ Department of Medicine, Rutgers Cancer Institute of New Jersey, New Brunswick, NJ, United States; ^2^ One Patient One Cure, Boston, MA, United States; ^3^ Department of Biotechnology, College of Basic Medical Sciences, Dalian Medical University, Dalian, Liaoning, China; ^4^ Department of Immunology, University of Texas (UT) Southwestern Medical Center, Dallas, TX, United States; ^5^ Child Health Institute of New Jersey, Rutgers Robert Wood Johnson Medical School, New Brunswick, NJ, United States

**Keywords:** tumor microenvironment, immunological niches, immune evasion, biomarkers, immunotherapy resistance

## Introduction

Immunological niches in tumor microenvironments (TMEs) are spatially organized functional units in which tumor cells interact with immune and stromal components via dynamic molecular networks (1). These specialized microdomains exhibit dual functionality: while they are capable of supporting anti-tumor immunity through immune cell activation, they frequently evolve immunosuppressive properties that enable immune evasion and therapy resistance (2). Emerging evidence reveals that niche characteristics significantly influence immunotherapy responses, with distinct spatial architectures associated with treatment sensitivity or resistance across cancer types (3). Understanding these regulatory mechanisms provides critical insights for developing strategies to therapeutically reprogram immunosuppressive niches.

## Decoding niche formation: cellular and molecular architects

Immunological niches within tumors emerge through coordinated interactions between three fundamental components: (1) tumor cells displaying antigenic and metabolic heterogeneity, (2) immune populations (CD8+ T cells, tumor-associated macrophages [TAMs], and dendritic cells [DCs]), and (3) stromal elements (cancer-associated fibroblasts [CAFs], the extracellular matrix [ECM], and the vasculature). These components communicate through cytokine-chemokine networks (e.g., IFN-γ-mediated immune activation versus TGF-β-driven suppression) and direct cell-contact signals (e.g., PD-1/PD-L1 checkpoint interactions) to collectively regulate anti-tumor immunity (2, 4).

At the cellular level, stromal components - particularly CAFs - have been identified as key architectural regulators (5). Single-cell multi-omics approaches have enabled the precise characterization of CAF heterogeneity. For instance, in colon adenocarcinoma, an 11-gene CAF signature effectively stratified patients into high- and low-risk groups, with the high-risk group exhibiting greater immune infiltration yet paradoxically lower drug sensitivity (Zhang et al.). Similarly, in triple-negative breast cancer (TNBC), Ding et al. established a prognostic model in which high-risk patients showed increased stromal CAF and endothelial cell infiltration and poorer clinical outcomes.

At the molecular level, pathway activation analyses reveal critical mediators of niche functionality (6). A pan-cancer study demonstrated that EGFR pathway activation drives immunotherapy resistance through an EGFR-related gene signature (EGFR.Sig), which correlates with elevated exhaustion markers (TIM-3 and LAG-3) and immunosuppressive ligands (PD-L1 and CD47) (Ye et al.). The convergence of these molecular and cellular pathways underscores the need for multidimensional therapeutic approaches targeting niche formation at multiple levels, a strategy that may help overcome therapeutic resistance and improve clinical outcomes.

## Biomarkers: from niches to clinical tools

Beyond mechanistic insights, translating immunological niches into clinically actionable biomarkers has become a critical frontier in precision oncology (7). Systemic inflammation indices derived from peripheral blood have demonstrated clinical utility as non-invasive niche proxies. As demonstrated in our Research Topic, these indices provide valuable insights for clinical prognosis and treatment strategies. For example, in esophageal cancer, the systemic immune-inflammation index (SII) was identified as an independent prognostic factor for recurrence-free survival after esophagectomy (Tan et al.). In breast cancer, an elevated preoperative systemic inflammation response index (SIRI) was found to serve as an independent risk factor for disease-free survival (Li et al.). Similarly, in locally advanced cervical cancer, the pan-immune-inflammation value (PIV) was identified as a robust and independent prognostic factor significantly correlated with both overall survival and disease-free survival (Yan et al.).

Niche-informed molecular subtyping is transforming patient stratification. In acute myeloid leukemia, molecular subtyping based on ligand-receptor (LR) pairs has unveiled distinct immune landscapes and prognostic differences. A scoring model termed LR.score was established to stratify patients by survival risk and reflect the degree of T-cell dysfunction, offering insights into niche-driven immune dysregulation and therapy resistance (Fu et al.). In melanoma, aryl hydrocarbon receptor (AhR)-related gene signatures (MAP2K1, PRKACB, KLF5, and PIK3R2) have been identified as potential prognostic tools strongly associated with immune infiltration and tumor progression, providing a robust prognostic framework and potential therapeutic targets (Li et al.).

These advances collectively represent a paradigm shift in cancer management, where niche-derived biomarkers are moving from research tools to clinical implementation. By capturing the complex interplay between tumor cells and their microenvironment, these biomarkers enable more precise patient stratification and treatment selection (8).

## Therapeutic strategies: rewriting niche rules

Although biomarkers yield valuable insights into niche dynamics, converting these findings into effective treatments remains a major challenge. To bridge this gap, therapeutic development is increasingly directed at reprogramming the tumor–immune interface by modulating niche biology through diverse approaches (9).

Targeting immune-related molecules remains a well-established and effective therapeutic approach. For example, RAC3 has been shown to drive tumor aggressiveness and immune evasion in bladder cancer, positioning it as a potential dual biomarker and therapeutic target (Gao et al.).

Stromal remodeling strategies are also gaining prominence. Evidence of clinical translation is already seen in the success of anlotinib (an anti-angiogenic agent) combined with anti-PD-L1 therapy in treating high-grade serous ovarian cancer. This combination therapy inhibits angiogenesis and enhances immune infiltration, while simultaneously reinvigorating exhausted T cells (Lan et al.), demonstrating the therapeutic potential of coordinated niche modulation.

Tumor metabolic regulators coordinate immune–tumor cell networks within the TME, and targeting these metabolic vulnerabilities is a promising yet clinically challenging therapeutic avenue (10). Ferroptosis inducers such as erastin enhance immunogenic cell death by releasing damage-associated molecular patterns (DAMPs) which recruit and activate immune cells, while lipid peroxides generated during ferroptosis may potentiate immune cell–mediated tumor killing (Liu et al.). In parallel, repurposing existing drugs such as metformin modulates the immune microenvironment, enhances the efficacy of immunotherapy and radiotherapy, and overcomes resistance in “cold” or refractory tumors, all while maintaining a favorable safety profile (Zhou et al.).

Notably, microbiome-targeted strategies are opening new therapeutic dimensions (11). TLR3 agonists, including Poly(I: C), have been shown to restore immune competence in colorectal cancer models with viral dysbiosis (Huang et al.), offering novel combination approaches.

Together, these diverse strategies demonstrate how targeting niche biology across stromal, metabolic, epigenetic, and microbial axes can help dismantle long-standing therapeutic barriers and expand the efficacy of cancer immunotherapy.

## Clinical challenges and future directions

Despite remarkable progress, significant challenges remain in translating niche biology into clinical practice (3). The dynamic nature of immunological niches complicates therapeutic targeting, as exemplified by cellular senescence transitioning from tumor-suppressive (p53/p21/p16-mediated) to pro-tumorigenic SASP states during cancer progression (Chen et al.). This plasticity necessitates precisely timed interventions and robust biomarkers to identify optimal treatment windows.

Unexpected systemic effects further complicate therapeutic development, with immune checkpoint inhibitors causing immune-related adverse events such as myositis in up to 30% of patients (Ma et al.). Emerging monitoring tools, including impedance myography and advanced serum biomarkers (Ma et al.), offer potential mitigation through early detection.

Technological innovations will be crucial for addressing niche complexity. Multiplex immunohistochemistry, spatial transcriptomics, and AI-enhanced image analysis are enabling high-resolution mapping of niche organization and evolution. These tools are particularly valuable in elucidating resistance mechanisms and tailoring personalized therapeutic strategies (12, 13). Integrating these technologies into clinical trials will be vital for translating niche biology into individualized cancer care.

## Conclusion

The formation and regulation of immunological niches within the TME represent both challenges and opportunities in cancer therapy. As evidenced throughout this Research Topic, targeting niche components—including immune, stromal, metabolic, and microbial elements—can enhance therapeutic precision and improve patient outcomes. Looking forward, a deeper mechanistic understanding and integrative translational efforts are indispensable to transforming niche biology from conceptual frameworks into clinically actionable strategies for precision oncology.
